# The biological impact of deuterium and therapeutic potential of deuterium-depleted water

**DOI:** 10.3389/fphar.2024.1431204

**Published:** 2024-07-22

**Authors:** Jiao Qu, Yufei Xu, Shuang Zhao, Ling Xiong, Jing Jing, Su Lui, Juan Huang, Hubing Shi

**Affiliations:** ^1^ Institute of Breast Health Medicine, State Key Laboratory of Biotherapy, West China Hospital, Sichuan University and Collaborative Innovation Center, Chengdu, China; ^2^ Department of Radiology, West China Hospital, Sichuan University, Chengdu, China

**Keywords:** deuterium, deuterium oxide, deuterium-depleted water, living systems, neoplasms, neuroprotective effect, antioxidants

## Abstract

Since its discovery by Harold Urey in 1932, deuterium has attracted increased amounts of attention from the scientific community, with many previous works aimed to uncover its biological effects on living organisms. Existing studies indicate that deuterium, as a relatively rare isotope, is indispensable for maintaining normal cellular function, while its enrichment and depletion can affect living systems at multiple levels, including but not limited to molecules, organelles, cells, organs, and organisms. As an important compound of deuterium, deuterium-depleted water (DDW) possess various special effects, including but not limited to altering cellular metabolism and potentially inhibiting the growth of cancer cells, demonstrating anxiolytic-like behavior, enhancing long-term memory in rats, reducing free radical oxidation, regulating lipid metabolism, harmonizing indices related to diabetes and metabolic syndrome, and alleviating toxic effects caused by cadmium, manganese, and other harmful substances, implying its tremendous potential in anticancer, neuroprotective, antiaging, antioxidant, obesity alleviation, diabetes and metabolic syndrome treatment, anti-inflammatory, and detoxification, thereby drawing extensive attention from researchers. This review comprehensively summarizes the latest progress in deuterium acting on living organisms. We start by providing a snapshot of the distribution of deuterium in nature and the tolerance of various organisms to it. Then, we discussed the impact of deuterium excess and deprivation, in the form of deuterium-enriched water (DEW) and deuterium-depleted water (DDW), on living organisms at different levels. Finally, we focused on the potential of DDW as an adjuvant therapeutic agent for various diseases and disorders.

## Introduction

As a stable and nonradioactive isotope of hydrogen, deuterium, denoted by the symbols ^2^H or D, is relatively rare in nature, with a deuterium-to-hydrogen (D/H) ratio of approximately 1:6,600, resulting in a deuterium abundance close to 150 ppm (0.015 atomic%) (Urey et al., 1932; Somlyai et al., 1993; Wang et al., 2013; Pirali et al., 2019; Di Martino et al., 2023). Deuterium differs from protium (^1^H) by bearing an added neutron in its nucleus, resulting in different atomic masses (twice that of hydrogen) and nuclear spins, distinct physicochemical properties, and disparate biochemical reactions, namely, isotopic effects (Mosin and Ignat, 2015; Wang et al., 2015; Zachleder et al., 2018; Pirali et al., 2019; Somlyai et al., 2020; Kopf et al., 2022). Existing evidence suggests that deuterium is a natural cell growth regulator that can control the balance between mitochondrial oxidation and reduction (Zhang et al., 2020), and a naturally occurring deuterium concentration and stable D/H ratio are vital conditions for maintaining the normal growth rate and signal transduction of the cell, as well as regulating the physiological functions of living systems (Somlyai et al., 1993; Somlyai et al., 1998; Somlyai et al., 2016; Somlyai et al., 2020). It can be hypothesized that cells possess a sub-molecular regulatory system (SMRS) which influences cellular genetic and biochemical processes through the actual D/H ratio (Somlyai et al., 2020), and two concurrent events can affect the intracellular D/H ratio: one is the binding of growth hormones to their receptors, which activates the H^+^ transport system, with certain enzymes or proteins (e.g., H^+^-ATP_ase_ and Na^+^/H^+^ exchanger) involved exhibit strong hydrogen/deuterium discrimination properties and preferentially or selectively consume hydrogen ions, increasing the intracellular D/H ratio, which is crucial for cell division; the other is related to the terminal complex of the electron transport chain in the properly working mitochondria that reduces molecular oxygen to deuterium-depleted water (DDW), thereby decreasing the D/H ratio within the cell and inhibiting cell growth (Perona and Serrano, 1988; Kotyk et al., 1990; Somlyai et al., 1993; Somlyai et al., 2016; Somlyai et al., 2020). In cancer cells, however, the impaired mitochondrial function affects the production of deuterium-depleted metabolic water, which may cause the cells more easily reach the D/H ratio required to trigger cell division.

Deuterium is mostly available as deuterium oxide (D_2_O), commonly known as “heavy water”, and also exists as HDO in aqueous solutions, in smaller content (Mosin and Ignat, 2015; Mosin and Ignatov, 2016; Zachleder et al., 2018; Kopf et al., 2022). Due to the substantial difference in atomic mass between D and H, D_2_O differs from H_2_O in many physical and chemical properties, such as density, viscosity, specific gravity, melting point and boiling point, as well as possessing deuterium bonds that are much stronger than the hydrogen bonds in H_2_O (PINSON, 1952; Uemura et al., 2002; Mosin and Ignat, 2015; Vergara et al., 2018; Zachleder et al., 2018; Kampmeyer et al., 2020). In chemical reactions, the rate at which chemical bonds containing deuterium split is significantly slower than that of bonds containing hydrogen (Somlyai et al., 2016). These differences have a pronounced impact on the structural and functional metabolism of cells. Specifically, the biological effects of D_2_O on living organisms are primarily attributed to its “solvent isotope effect” and “deuterium isotope effect”, with the former related to the properties of D_2_O itself and primarily affecting the structure of water and macromolecules, while the latter resulting from the replacement of hydrogen bonds with deuterium bonds in biological molecules (Vergara et al., 2018; Kampmeyer et al., 2020; Chen, 2022). Both the solvent isotope effect and the deuterium isotope effect can exert extensive and complex impacts on living organisms, such as affecting the conformation of biomolecules by altering their solubility and structural stability, leading to changes in cell growth and division, protein rigidity, gene expression, fatty acid composition, enzyme activity, ATP production, and organ metabolism, thereby influencing the physiological functions of organisms (Vergara et al., 2018; Zachleder et al., 2018).

The mass of water with a volume fraction of deuterium lower than the naturally occurring D concentration is referred to as DDW (Wang et al., 2013; Rehakova et al., 2016; Rasooli et al., 2019; Wu et al., 2020); conversely, it is termed deuterium-enriched water (DEW). Previous *in vitro* and *in vivo* experiments commonly used D_2_O, in the form of DDW or DEW, to alter the D/H ratio in cells or organisms, thereby exploring the effects of increased or decreased deuterium concentrations on life systems from multiple perspectives. As early as 1993, Somlyai et al. mentioned the potential efficacy of DDW in inhibiting tumor growth in xenotransplanted mice (Somlyai et al., 1993). Since then, numerous publications have explored the antitumoral effect of DDW both *in vitro* and *in vivo*, and prospective and retrospective clinical studies have also aimed to investigate the potential of DDW to extend the survival of cancer patients and to alleviate subjective symptoms (Cong et al., 2010; Kovács et al., 2011; Wang et al., 2013; Somlyai et al., 2023). Subsequent studies have also shown a positive correlation between the D-content in drinking water and human susceptibility to depression, and found the effects of DDW on stimulating long-term memory in rats, counteracting oxidative stress-mediated cellular damage by inducing endogenous antioxidants in cells, reversing the shortening of lifespan induced by manganese (Mn) in *Caenorhabditis elegans*, alleviating diet-induced obesity and related metabolic damage in rat models, and regulating serum parameters associated with diabetes and metabolic syndrome, suggesting that DDW may play an important role in the treatment or prevention of various diseases or disorders, such as depression, aging, obesity, and diabetes (Ávila et al., 2012; Mladin et al., 2014a; Strekalova et al., 2015; Halenova et al., 2019; Somlyai et al., 2020; Wu et al., 2020; Zlatska et al., 2020).

**FIGURE 1:**
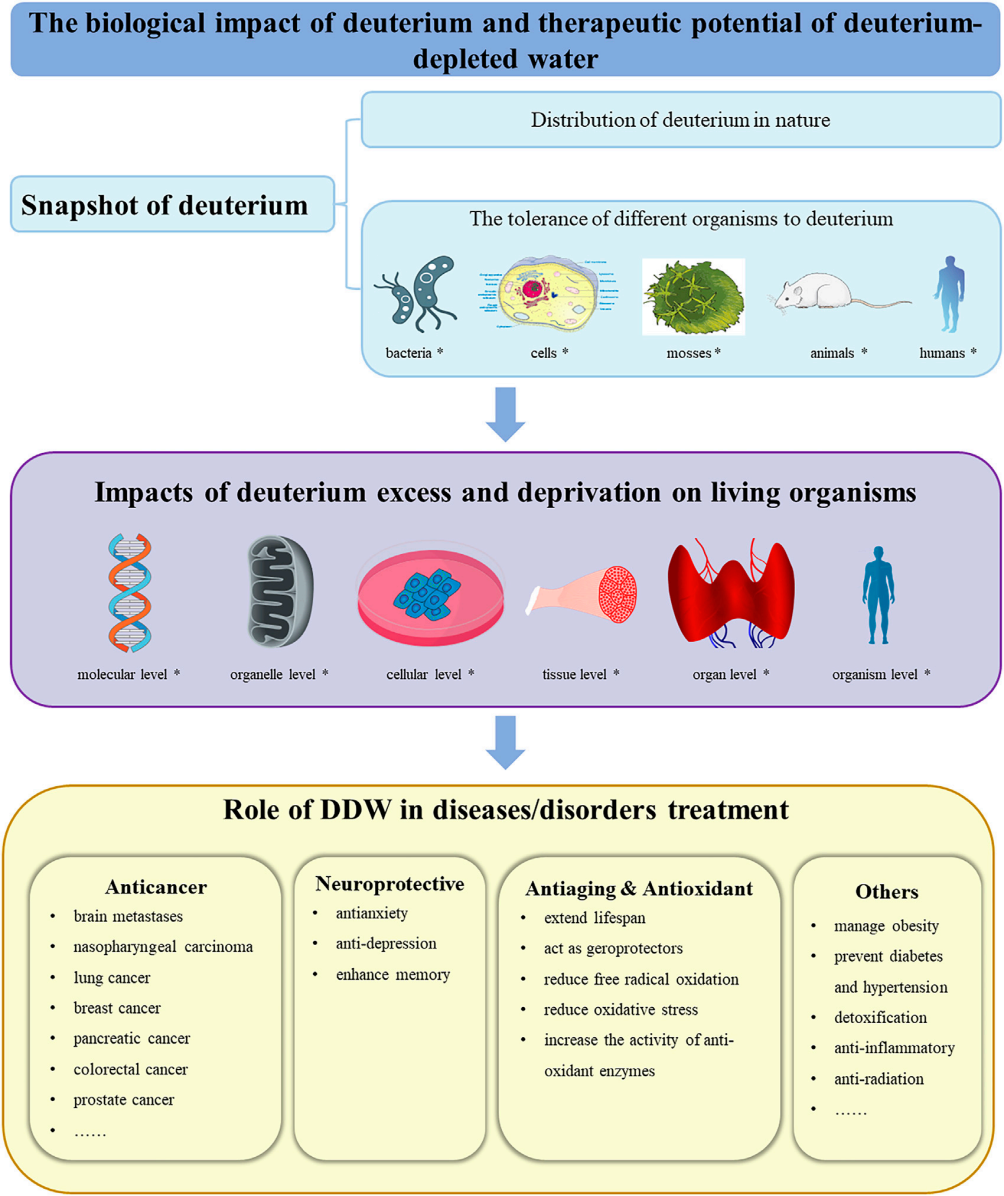
A snapshot of this review. First, an overview of deuterium, including the distribution of deuterium in nature and the tolerance of different organisms to deuterium, was introduced. Subsequently, the effects of deuterium excess and deprivation on organisms at various levels were discussed. Finally, the potential of deuterium-depleted water in assisting the treatment of various diseases or disorders was elaborated. *: Icons downloaded from the open-source libraries Bioicons (https://bioicons.com/) and PinClipart (https://www.pinclipart.com/); DDW: deuterium-depleted water.

To comprehensively understand the biological impact of deuterium and therapeutic potential of deuterium-depleted water, we start by briefly overviewing the distribution of deuterium in nature and the tolerance of different organisms to it. Next, we focus on the impacts of deuterium excess or depletion on living organisms at the molecular, organelle, cellular, tissue, organ and organism levels. Finally, taking into account the increasing attention given to DDW as a potential adjuvant therapeutic agent for various diseases and cancer in particular, we elaborate in detail on the specific properties of DDW, such as its anti-cancer, neuroprotective, antiaging, antioxidant, hypoglycemic, and anti-inflammatory effects, aiming to provide new perspectives for the treatment of these disorders or diseases (Figure 1). While the content of deuterium-labeled molecular probes used for MRI metabolic imaging, the applications of deuterium-labeled compounds as tracers in ecology, proteomics, metabolomics, etc., as well as the development and application of deuterated drugs in medicinal chemistry (Atzrodt et al., 2018; Pirali et al., 2019; Kopf et al., 2022; Li et al., 2022; Di Martino et al., 2023; Liu et al., 2023), are beyond the scope of the present review.

## Distribution of deuterium in nature and the tolerance of organisms to it

Deuterium, produced by nucleosynthesis during the Big Bang, exists not only in stars but also on Earth, with variations in its distribution between marine and nonmarine regions (Epstein et al., 1976; Mosin and Ignat, 2015; Di Martino et al., 2023). Natural water is a multicomponent mixture composed of water isotopologues consisting of isotopes of hydrogen (^1^H, ^2^H, ^3^H) and oxygen (^16^O, ^17^O, ^18^O) (Strekalova et al., 2015; Syroeshkin et al., 2019; Zlatskiy et al., 2020), in which the deuterium content varies geographically and is influenced by many factors, such as altitude, latitude, humidity, and seasonal temperature (Friedman et al., 1963; Friedman and Smitgi, 1970; Kendall and Coplen, 2001; Strekalova et al., 2015; Bayrak et al., 2022). In areas close to the North and South poles, inland continental, and high-altitude regions, the concentration of deuterium in precipitation is relatively low (Friedman and Smitgi, 1970; Somlyai et al., 2016). As a result, the abundance of deuterium in natural water resources on Earth varies from D/H = 90 ppm in glacial water to D/H = 200 ppm in deep sea water (Syroeshkin et al., 2018), while in regular water, it is approximately 150 ppm (exceeding 16 mM/L) (Kovács et al., 2011). The supply of deuterium in cells and tissues mainly comes from two sources: one is external intake, i.e., deuterium ingested from natural water, and the other is intrinsic water generated by the transformation of metabolites, including the reduced form of nicotinamide adenine dinucleotide (NAD) in the mitochondrial respiratory chain (Yaglova et al., 2023a). Before being oxidized by mitochondria, the hydrogen and deuterium in intrinsic water are bound to organic molecules such as lipids, carbohydrates, and proteins, which exhibit significant differences in their deuterium content, resulting in varying deuterium levels in their metabolic water. For instance, a diet rich in carbohydrates produces metabolic water with a D-concentration close to 155.75 ppm, while complex molecules like lipids have lower deuterium content, leading to a reduced deuterium concentration in the metabolic water they produce (Somlyai et al., 2020; Somlyai et al., 2022). Therefore, the deuterium content in the human body and other living organisms vary slightly based on the source of drinking water, dietary preferences and different stages of the life, typically ranging from 12–14 mM/L (Wang et al., 2013; Li and Snyder, 2016a; Rehakova et al., 2016; Somlyai et al., 2016; Yavari and Kooshesh, 2019; Somlyai et al., 2020; Somlyai et al., 2022; Yaglova et al., 2023a). It was only recently discovered that the concentration of deuterium in bone collagen from seals reached 310 ppm, which is higher than any previously reported D concentration in biological samples (Gharibi et al., 2022).

The tolerance of living systems to deuterium exhibits species specificity (Table 1). Generally, the lower the evolutionary organization level of an organism is, the easier it is to adapt to the existence of deuterium in the culture medium (Mosin and Ignat, 2015). Some mosses and algae, such as Funaria hygrometrica and Chlamydomonas reinhardtii, can grow in media containing 90%–99.6% D_2_O and exhibit strong deuteration patterns, making them ideal organisms for producing valuable deuterium-labeled compounds (CRESPI and KATZ, 1959; Vergara et al., 2018; Kselíková et al., 2021). Other prokaryotes and lower eukaryotes have also been reported to be capable of growing in concentrations of D_2_O as high as 99.6–99.8 atom% (Zachleder et al., 2018). Many kinds of bacteria, yeasts, and molds even tolerate fully deuterated medium (Borek and Rittenberg, 1960; Katz and Crespi, 1966; de Carli et al., 2020; Kampmeyer et al., 2020; Kleemann et al., 2020). While some plant cells can develop normally in up to 75% DEW (Mosin and Ignatov, 2014; Mosin and Ignat, 2015; Zhang et al., 2020), most mammalian cells are intolerant to high concentrations of D_2_O, as it is noxious or lethal to them (Kushner et al., 1999; Mosin and Ignat, 2015; Zachleder et al., 2018). It was reported that D_2_O at concentrations as low as 30%–40% can significantly arrest or delay cell division, and a concentration reaching 75% completely inhibited the mitosis in sea urchin eggs (GROSS and SPINDEL, 1960). When exposed to D_2_O at a concentration of up to 50%, cells can survive, but a further increase in the deuterium concentration could lead to cellular degradation and death (Rothstein et al., 2006).

**TABLE 1:** The tolerance of certain organisms to deuterium.

Organisms	Tolerable D_2_O concentration (%)
Some bacteria and algae (Zachleder et al., 2018; de Carli et al., 2020; Kampmeyer et al., 2020)	99.4–100
Some yeasts and fungi (Zachleder et al., 2018; Kampmeyer et al., 2020)	90-99.8
Some protozoa (Zachleder et al., 2018)	70–100
Some plant cells (Mosin and Ignatov, 2014; Mosin and Ignat, 2015; Zhang et al., 2020)	75
Mammalian cells (Mosin and Ignatov, 2014; Mosin and Ignat, 2015; Zachleder et al., 2018; Zhang et al., 2020)	30–50
Mice, rats, dogs, fish, tadpoles, flat-worms, and *drosophila* (Kushner et al., 1999; Zachleder et al., 2018; Kampmeyer et al., 2020)	25

Animals such as mice, rats, dogs, fish, tadpoles, flatworms, and *drosophila* can survive in an environment with a deuterium content of 25%, albeit at the cost of decreased reproductive potential (Kushner et al., 1999; Zachleder et al., 2018). It has been reported that replacing hydrogen with deuterium by administering 25% D_2_O in the drinking water significantly impaired the reproductive potential of female mice, manifested as a decreased number of pregnancies carried close to term, an increased incidence of completely nonviable litters, and a reduced survival rate of newborn mice (Czajka and Finkel, 2006). In another study, maintaining serum deuterium concentrations at 20%–34% was considered to result in azoospermia or sterility in male mice (HUGHES and CALVIN, 1958; Hughes et al., 1959; AMAROSE and CZAJKA, 1962). And higher concentrations of D_2_O feeding can soon lead to weakness, abnormal changes in various serum laboratory indices, neuromuscular disturbances, and damage or pathological changes in organs such as the heart, liver, spleen, kidneys, testes, and salivary glands, although some physiological damage appears to be reversible (KATZ et al., 1957; CZAJKA et al., 1961; BACHNER et al., 1964; Jones and Leatherdale, 1991; Kushner et al., 1999; Czajka and Finkel, 2006; Zachleder et al., 2018). In a study by Uemura et al., replacing approximately 30% of the H_2_O in the body with D_2_O even significantly decreased the survival rate of rats (Uemura et al., 2002). Consistent with this finding, other studies have also shown that replacing more than 35% of the water in the body with heavy water is incompatible with the long-term survival of mice (KATZ et al., 1957; Altermatt et al., 1988), and extremely high concentrations of D_2_O (approximately 90%) in the environment can rapidly kill fish, tadpoles, flatworms, and *drosophila* (Kushner et al., 1999; Klein and Klein, 2013). For humans, a single oral dose of 140–250 g of 99.5% D_2_O can elevate the D-content in body water to 0.5%, which may result in transitory vertigo, and possibly associated with interference of the specific gravity of the vestibular fluid by heavy water (Jones and Leatherdale, 1991; Kleemann et al., 2020). Some also believe that a concentration of D_2_O up to 23% in body fluids, in the short term, does not pose toxicity (Wallace et al., 1998; Kushner et al., 1999). However, the effects of long-term exposure to high concentrations of D_2_O have not been determined.

## Effects of deuterium excess and depletion on living organisms

As a solvent and an indispensable active component in biomolecular systems, water provides a liquid phase that enables reactants to come into contact, thereby facilitating chemical reactions while also maintaining the functional structure of various biomolecules, such as proteins, nucleic acids, and lipids (Kampmeyer et al., 2020). The normal D/H ratio in water plays a crucial role in ensuring the passage and kinetics of many biochemical reactions and regulating the energy metabolism and functional activity of mitochondria, and thus it is of great significance in maintaining the normal growth of living systems (Kselíková et al., 2019; Zlatskiy et al., 2020). In the experiments conducted by Somlyai et al., the inhibited effects of DDW on the growth of animal cell lines, and the prolonged survival accompanied by the fact that in 59% of the tumorous mice experienced tumor regression and disappearance because of the treatment with DDW (30 ppm) suggested that a certain concentration of deuterium is essential for normal cell growth, particularly the proliferation of tumor cells (Somlyai et al., 1993). It is hypothesized that the cell cycle regulatory system is capable of recognizing changes in the D/H ratio in some way, and when this ratio reaches a certain threshold, it triggers the molecular mechanism which promotes cell to enter the S phase (Somlyai et al., 1993; Wang et al., 2013). While changes in deuterium concentration can impact the stability, function, dynamics, and interactions of certain biomolecules, as well as affecting the strength of some structures, such as hydrogen bonds, hydrophobic interactions, and carbon-hydrogen bonds, which are considered crucial driving factors in various biological processes (Chen, 2022). From an evolutionary perspective, living organisms have adapted to natural water with a constant D/H ratio. Hence, life processes are highly sensitive to changes in deuterium concentration. Even slight increases or decreases in the D/H ratio in water may have profound effects on the physiological processes and biological activities of living organisms at the molecular, organelle, cellular, tissue, organ and even whole organism levels (GROSS and SPINDEL, 1960; Zubarev, 2011; Boros et al., 2016; Dzhimak et al., 2018; Basov et al., 2019a; Basov et al., 2019b; Zlatskiy et al., 2020; Kozin et al., 2021; Kravtsov et al., 2021).

### Deuterium excess effects on organisms

Generally, the enrichment of heavy stable isotopes tends to slow the rate of chemical and biochemical reactions, which is known as the kinetic isotope effect, and from a biological angle, this reduction in reaction rates implies the slowing of the growth rate (Li and Snyder, 2016a; Xie and Zubarev, 2017; Kselíková et al., 2021). In fact, multiple studies have confirmed this inference. The extensive replacement of hydrogen by deuterium has resulted in several negative effects on living systems, including but not limited to: 1. Disrupting cell division mechanisms, impairing cell cycle progression, inhibiting cell differentiation, and slowing cellular metabolism and growth (KATZ et al., 1957; MANSON et al., 1960; Takeda et al., 1998; Zubarev, 2011; Mosin and Ignat, 2015; Zlatska et al., 2020; Kselíková et al., 2021); 2. Reducing protein and nucleic acid synthesis, as well as affecting the structure of DNA (Jones and Leatherdale, 1991; Takeda et al., 1998; Zubarev, 2011; Abilev et al., 2020; Wu et al., 2020); 3. Altering the kinetics rates and conformation of enzymes, thereby affecting their activity and depressing tissue metabolism (KATZ et al., 1957; Wu et al., 2020); 4. Increasing the mutation frequencies of certain bacteriophages and bacteria (DE GIOVANNI, 1961; Konrad, 2006; Ajibola et al., 2021); 5. Causing organ damage, affecting the reproductive potential and shortening the lifespan of animals (Barbou et al., 1938; Hughes et al., 1959; AMAROSE and CZAJKA, 1962; BACHNER et al., 1964; Czajka and Finkel, 2006; Ávila et al., 2012). It was reported that deuterium at concentrations higher than natural levels (100- to 1000-fold) has significant, mainly toxic, effects (Somlyai et al., 2020; Ajibola et al., 2021; Somlyai et al., 2021a). The excessive D not only inhibit cell growth and division by affecting microscopic kinetics but also increase protein rigidity, slow down ATP production, influence gene expression, fatty acid composition, and hydration of membrane lipids, as well as impact the function of Ca^2+^ and Cl^−^ channels through osmotic-like stress (Zachleder et al., 2018). Deuterium-enriched water with D concentrations up to 99% almost completely inhibited mitochondrial respiratory chain reactions (Syroeshkin et al., 2018). Excessive deuterium also negatively impacts cellular metabolism, leading to physiological, morphological, and cytological alterations in cells (Mosin and Ignatov, 2014; Ignatov et al., 2015), including but not limited to decreased cell proliferation and metabolic activity, inhibited cell cycle, prolonged cell doubling time, and altered cell morphology (Mosin and Ignat, 2015; Zlatska et al., 2018; Kleemann et al., 2020; Kozin et al., 2021; Kselíková et al., 2022). Kampmeyer et al. demonstrated that high concentrations of D_2_O lead to alterations in glucose metabolism and growth inhibition in *Schizosaccharomyces pombe*. After long-term incubation in D_2_O at high concentrations, the cells underwent noticeable gross morphological changes, characterized by thickened cell walls and abnormal cytoskeletons. Further transcriptomic and gene analysis indicated that D_2_O substitution for water activated the heat shock response pathway and the cell integrity pathway (Kampmeyer et al., 2020). The partial reason for the inhibition of cell growth, proliferation, and division by high concentrations of deuterium is the impact of excessive deuterium on microtubule dynamics (Altermatt et al., 1988; Kampmeyer et al., 2020). Furthermore, the substitution of H_2_O by D_2_O in cells could affect the reaction kinetics and macromolecular structure, resulting in the so-called “solvent isotope effect”.

Despite the adverse impacts and toxic effects of deuterium excess on biological systems to some extent, at appropriate concentrations, excess deuterium may produce desirable protective effects. For instance, a slight enrichment of deuterium (350 ppm) in water has been shown to accelerate the growth of human cells by reducing the production of reactive oxygen species (ROS) in mitochondria (Zhang et al., 2020). Supplementation with 10% D_2_O in the diet of age-related hearing loss (ARHL) mice can effectively slow the metabolic rate and reduce the production of endogenous oxidative stress in the cochlea, thereby impeding the progression of ARHL (Hou et al., 2022). Achieving moderate levels of deuteration by adding 7.5% and 15% D_2_O to the regular diet significantly extended the mean lifespan of *Drosophila melanogaster* without impairing fecundity (Hammel et al., 2013). Evidence also suggested that D_2_O at a concentration of 25% could act as a calcium channel blocker to normalize calcium uptake in vascular smooth muscle, thereby preventing hypertension (Vasdev et al., 1990a; Vasdev et al., 1990b; Vasdev et al., 1991). The possibility of D_2_O as a therapeutic agent against malignant tumors was also explored in previous studies, which indicated that the combined use of 30% D_2_O and methotrexate significantly prolonged the survival of tumor-bearing mice; 40% D_2_O inhibited the growth of carcinoma and lymphosarcoma in mice; and D_2_O at concentrations of 5%–50% effectively inhibited the growth of various human digestive organ cancer cell lines, including AsPC-1, BxPC-3, PANC-1, HepG2, Colo205, and KATO-3 (Barbou et al., 1938; Laissue et al., 1982; Takeda et al., 1998; Hartmann et al., 2005). In addition, an organ preservation solution containing 30% D_2_O can alleviate hepatic ischemia and reperfusion injury (IRI) in rats by inhibiting edema, improving hepatic oxygen delivery and utilization, and preventing cytoplasmic Ca^2+^ overload and the subsequent mitochondrial damage, cellular cytoskeletal disruption, apoptosis and necrosis (Shimada et al., 2019). On the other hand, D_2_O has also been reported to have radioprotective effects, as demonstrated in a previous study where mice given 30% deuterated drinking water for 12 days before irradiation exhibited significantly reduced post-irradiation mortality compared to that of control mice drinking regular water (Laissue et al., 1983). In a dosage-dependent manner, D_2_O also serves as a metabolic modifier to promote the longevity of yeast (Li and Snyder, 2016a; Li and Snyder, 2016b). Furthermore, 60% D_2_O can remarkably decrease the release of dialysate myoglobin induced by coronary occlusion and reperfusion and chemical hypoxia, which offers protection against myocardial reperfusion injury in rats and may exert beneficial effects on coronary revascularization therapies (Ishikawa et al., 2019).

### Deuterium depletion effects on organisms

Compared to the detrimental and toxic effects caused by excess deuterium on cells, the biological effects of a reduced deuterium content are relatively positive and primarily manifest as regulating cellular respiration, increasing cellular energy, and promoting the growth of normal cells (Wang et al., 2013; Bayrak et al., 2022). It has been reported that media containing DDW at concentrations of 100 ppm, 75 ppm, and 50 ppm intriguingly promoted the growth of normal pre-osteoblast MC3T3-E1 cells (Wang et al., 2013). A prior study also suggested that DDW (D/H = 30 ± 2 ppm) based growth medium could facilitate colony formation in early passages and increase the proliferation of human dermal fibroblasts *in vitro*, supporting the beneficial effects of DDW (Syroeshkin et al., 2018). Lowering the deuterium concentration in water can also alter the metabolic rate of mitochondria and the mitochondrial oxidation function (Pomytkin and Kolesova, 2006; Boros et al., 2016; Yaglova et al., 2023b). In addition, the intake of DDW was reported to significantly reduce the number of single-stranded DNA breaks and enhance the efficiency of cellular defense systems (Dzhimak et al., 2016; Yaglova et al., 2023a).

At the organ level, reducing the deuterium concentration in the body significantly affect the secretion activities of the thyroid and pituitary glands, thereby influencing the endocrine patterns (Yaglova et al., 2023b; Yaglova et al., 2023c). Researches by Yaglova et al. indicated that not only a rapid decrease in deuterium content in the body resulting from the consumption of DDW can affect the function of the pituitary-thyroid gland axis but also a gradual decrease in body deuterium content can impact the function of the entire hypothalamic-pituitary-thyroid axis (Yaglova et al., 2021a; Yaglova et al., 2023c). Additionally, DDW enables thermoregulation of rats by reducing the amplitude of temperature fluctuations and enhancing temperature stability, which may be attributed to the impact of DDW on the synthesis of some fluid factors that control thermogenesis, such as hormones (Yaglova et al., 2021b).

Research on the impact of deuterium depletion on living organisms also primarily focuses on utilizing DDW as adjunctive therapy for certain diseases and disorders, such as tumors, depression, aging, hypertension, diabetes, and metabolic syndrome. These findings will be detailed in the section “Role of DDW in diseases/disorders treatment” below.

## Role of DDW in diseases/disorders treatment

Deuterium-depleted water in nature primarily exists in the ice and snow of the Earth’s polar regions and high mountains. It can also be obtained by reducing the deuterium concentration in natural water through specific methods such as distillation and electrolysis. The concentration of deuterium in the body correlates well with the deuterium levels in the environment; therefore, the deuterium depletion status in organisms can be obtained by DDW intake (Gyöngyi et al., 2013; Rehakova et al., 2016; Rasooli et al., 2019). According to preliminary studies, the consumption of DDW within a certain D-concentration range (25–135 ppm) is considered safe with no adverse events (Krempels et al., 2013; Somlyai et al., 2020; Somlyai et al., 2021a). The use of DDW for adjuvant disease treatment has become a research hotspot in recent years, and previous studies have described the significant value of DDW in areas such as anti-cancer, neuroprotective, antiaging, antioxidant, and antidiabetic effects.

### Anti-cancer effect of DDW

Three decades ago, a study by Somlyai et al. reported that 30–40 ppm DDW dramatically inhibited tumor growth in xenotransplanted mice and significantly increased the survival of tumor-bearing mice (Somlyai et al., 1993; Somlyai et al., 2010a). Following this groundbreaking research, numerous studies have been dedicated to exploring the potential of DDW as an anticancer agent in both *in vitro* and *in vivo* models involving but not limited to nasopharyngeal carcinoma, lung cancer, breast cancer, pancreatic cancer, colorectal cancer, melanoma, and prostate cancer (Gyöngyi and Somlyai, 2000; Hartmann et al., 2005; Somlyai et al., 2010b; Cong et al., 2010; Kovács et al., 2011; Gyöngyi et al., 2013; Wang et al., 2013; Chira et al., 2018; Yavari and Kooshesh, 2019; Boros et al., 2021; Somlyai et al., 2021b), opening a new strategic approach in cancer therapy. To date, many studies have confirmed the inhibitory effect of DDW on various tumor cell lines. It was reported that DDW at concentrations of 30–100 ppm significantly suppressed the growth of human MCF7 breast cancer cell lines, causing cell cycle arrest at the G1/S transition, reducing the number of cells in the S phase while increasing the number of cells in the G1 phase, and remarkably increasing the activities of superoxide dismutase (SOD) and catalase (CAT) enzymes (Yavari and Kooshesh, 2019). DDW at a concentration of 50 ppm was found to induce apoptosis in the human lung cancer A549 cell line (Cong et al., 2010). Besides, the inhibitory effect of DDW on lung cancer cells has also been confirmed *in vivo*, and it has been explained as possibly being related to the downregulation of several cancer genes, such as Kras, B-cell lymphoma 2 (Bcl2), and c-Myc, induced by DDW (Gyöngyi et al., 2013). In addition, DDW can significantly inhibit cell migration, which is considered an important step in tumor development and metastasis (Wang et al., 2013). In an *in vitro* experiment by Wang et al., DDW effectively suppressed the proliferation, migration and invasion of nasopharyngeal carcinoma cell lines by arresting the cell cycle, promoting the expression of quinone oxidoreductase-1 (NQO1), and downregulating the expression of proliferating cell nuclear antigen (PCNA) and matrix metalloproteinase 9 (MMP9) (Wang et al., 2013). Using a scratch assay, Syroeshkin et al. demonstrated the inhibitory effect of DDW on the amoeboid cell movement of the A549 and HT29 cell lines (Syroeshkin et al., 2018). Last but not least, the synergistic anti-cancer effects of DDW with antitumor agents, including 5-fluorouracil and the cytostatic compound methotrexate, have also been mentioned in previous studies (Yavari and Kooshesh, 2019).

Taken together, the ways in which DDW exerts its anti-cancer effects can be summarized as follows: 1. Suppressing the proliferation and division of tumor cells (Somlyai et al., 1999; Gyöngyi et al., 2013; Wang et al., 2013); 2. Inducing tumor cell apoptosis, cell cycle arrest, and the expression of cell cycle-related regulatory proteins (Cong et al., 2010; Gyöngyi et al., 2013; Wang et al., 2013); and 3. Inhibiting the expression of certain genes associated with tumor occurrence, development, and prognosis (such as H-Ras, p53, c-Myc, Kras, COX-2, and Bcl2) (Somlyai et al., 2010b; Gyöngyi et al., 2013; Kovács et al., 2022). Additionally, one possible mechanism by which DDW inhibits tumor cell proliferation is through the induction of an imbalance between the production and neutralization of reactive oxygen species (ROS) in mitochondria, which leads to cellular oxidative stress and thereby results in slowed cell growth and induction of apoptosis (Zhang et al., 2019; Bayrak et al., 2022). The possible reason for the inhibition of tumor cell growth by DDW may be due to changes in the D/H ratio, as cell growth and division require a certain concentration of deuterium to regulate the cell cycle, and a reduction in deuterium content prolongs the time needed to achieve an appropriate D/H ratio, thereby interfering with signal transduction pathways, slowing the growth of cancer cells, and promoting tumor regression (Somlyai et al., 1993; Somlyai et al., 2010b; Cong et al., 2010; Yavari and Kooshesh, 2019; Kovács et al., 2022).

Building upon the aforementioned preclinical studies, limited clinical trials were conducted, with results showing that DDW has effects such as extending the lifespan of patients, alleviating subjective symptoms, reducing tumor size, and preventing tumor metastasis and recurrence (Krempels et al., 2008; Kovács et al., 2011; Gyöngyi et al., 2013; Krempels et al., 2013; Boros et al., 2021; Somlyai et al., 2021b; Kovács et al., 2022; Somlyai et al., 2023) (Please refer to Table 2). In a randomized, double-blind phase II clinical trial involving of prostate cancer patients, DDW, as an adjunct to conventional therapy, significantly prolonged the 1-year survival rate of patients (Kovács et al., 2011). While most anticancer chemotherapy drugs have minimal effectiveness in treating malignant tumors of brain due to their inability to penetrate the blood brain barrier (BBB), DDW provides a new perspective for the adjuvant treatment of brain metastasis (BM) and glioblastoma multiforme (GBM), as confirmed in two prior studies in which DDW consumption in combination with conventional therapies noticeably prolonged the survival time of the lung cancer patients with BM and the GBM patients (Krempels et al., 2008; Wang et al., 2013; Somlyai et al., 2023).

**TABLE 2:** Clinical trials of DDW for antitumor effect.

Author	Years	Country	Study type	Tumor	No. Of patients	D-concentration (ppm)	Mainly result
Somlyai et al. (Somlyai et al., 2021b)	2021	Hungary	Retrospective	NSCLC	183	25–105	Compared to MST of historical control (8–12 months), the combination of DDW and conventional therapies increased the MST by six- and fivefold (61.9 and 48.4 months)
Somlyai et al. (Somlyai et al., 2023)	2023	Hungary	Retrospective	GBM	55	85, 65, 45, and 25	Conventional therapies supplemented with DDW achieved a longer MST (30 months) compared to the historical control (12.1–14.6 months)
Kovács et al. (Kovács et al., 2022)	2022	Hungary	Retrospective	Multiple tumors	204	105, 85, 65, 45, and 25	DDW consumption prevented tumor recurrence^*^
Kovács et al. (Kovács et al., 2011)	2011	Hungary	Prospective and Retrospective	PC^a^	113	85 ppm and 25–105	DDW prolonged MST (11.02 years) in patients with PC.
Krempels et al. (Krempels et al., 2008)	2008	Hungary	Retrospective	BM	4	25–95	The survival times of four lung cancer patients with BM who received DDW combined with conventional treatment were 26.6, 54.6, 21.9, and 33.4 months, respectively, which were noticeably longer than the median survival time (MST = 4–6 months) reported in previous literature
Gyöngyi et al. (Gyöngyi et al., 2013)	2011	Hungary	Retrospective	LC	129	25–105	The combination of DDW and conventional treatment extend the survival of LC patients, especially female patients with tumor overexpression of cancer-related genes (MST = 74.1 months)
Krempels et al. (Krempels et al., 2013)	2013	Hungary	Retrospective	BC	232	65–105	DDW consumption in addition to the conventional therapies prolonged the survival of BC patients (MST for early-stage BC: 217 months; MST for advanced BC: 52 months). And the MST for patients who received DDW therapy at least twice was 293 months, compared to 108 months for those who received a single DDW therapy
Boros et al. (Boros et al., 2021)	2021	Hungary	Retrospective	PC^b^	86	45, 65, and 85	The MST for patients consuming DDW parallel to the conventional therapies was 19.6 months, in comparison with 6.36 months for patients accept chemotherapy alone

BC: breast cancer, BM: brain metastases, D: deuterium, DDW: deuterium-depleted water, GBM: glioblastoma multiforme, LC: lung cancer, MST: median survival time, NSCLC: non-small cell lung cancer, No.: number, PA: pancreatic adenocarcinoma, PC^a^: prostate cancer, PC^b^: pancreatic carcinoma, *: The MST, was not calculable due to the extremely low death rate (13/204).

### The neuroprotective effects of DDW

Special attention should be paid to the neuroprotective effects of DDW, which are primarily manifested in its ability to alleviate anxiety and depression, as well as enhance long-term memory, among other aspects. Animal experiments conducted by Mladin et al. not only described the anxiolytic-like effect of DDW but also demonstrated its potential to improve long-term memory in rats, evidenced by an increase in the percentage of time and number of entries in the open arms of the elevated plus maze, and a significant reduction in reference memory errors in the radial arm maze for the DDW group, along with a decrease in the time needed to complete the test (Mladin et al., 2014a; Mladin et al., 2014b). Using epidemiological methods, Strekalova et al. investigated the relationship between the deuterium content of tap water and the incidence of depression in certain regions of the USA and found a positive correlation between the two, with a 1.8% increase in the prevalence of depression for every 10-ppm increase in deuterium concentration. These findings were also confirmed in subsequent animal experiment, which suggested that reducing deuterium intake potentially reversed the depressive-like state, altered sleep patterns in mice, and affected the gene expression associated with serotonergic neurotransmission (Strekalova et al., 2015).

The biological mechanisms underlying the neuroprotective effects of DDW still need further elucidation, and they are likely related to a series of biological and biochemical reactions triggered by the replacement of naturally occurring water with DDW, including the activation of proton transport through the membrane, an increase in the efflux of protons to the extracellular space, a consecutive increase in the intracellular (cytosolic) pH, the activation of acid-sensing ion channels (ASICs), and increased synaptic efficacy (Mladin et al., 2014a; Mladin et al., 2014b). In addition, the increased fluidity of cell membranes and the weakened rigid structure of phospholipid bilayers induced by DDW can also alter the distribution of neurotransmitter receptors and increase receptor affinity, as well as affect the permeability of the BBB, which promotes neural activity and alleviates symptoms of depression (Strekalova et al., 2015).

### Antiaging and antioxidant properties of DDW

The continuous increase in the proportion and duration of global population aging has imposed a heavy socioeconomic burden on humanity. In recent years, DDW has garnered special attention from researchers in the field of antiaging. In a previous study, compared with control rats ingesting plain water with a deuterium content of 150 ppm, presenile female rats (20–22 months old) that consumed DDW at a concentration of 46 ± 2 ppm for a period of 5 weeks exhibited signs of a restored estrous cycle, an improved coat state, and enhanced skin bactericidal power, providing experimental evidence for the geroprotector properties of DDW (Dzhimak et al., 2018). There was also additional evidence suggested that DDW at a concentration of 90 ppm significantly reversed the manganese (Mn)-induced aging in *C. elegans* by regulating the levels of DAF-16 (a lifespan-regulating factor signaling pathway) and restoring superoxide dismutase (SOD-3) levels, thereby extending its lifespan (Ávila et al., 2012). In light of the current free radical theory of aging, if these observations represent a widespread phenomenon in other organisms as well, especially in humans, it would open up a novel perspective for antiaging and longevity promotion.

Oxidative stress is widely recognized as an important pathogenic factor in aging and various diseases, such as cancer, neurodegenerative diseases, diabetes, hypertension, and atherosclerosis (Lin and Beal, 2006; Kajiyama et al., 2008; Wu et al., 2020; Bayrak et al., 2022). DDW achieves antioxidant effects by inducing endogenous antioxidants to counteract oxidative stress-mediated cellular injury (Wu et al., 2020). Accumulating evidence has shown that DDW has a significant impact on antioxidant indicators in the brain, serum, and liver (Kajiyama et al., 2008; Rasooli et al., 2019; Wu et al., 2020; Kravtsov et al., 2021; Bayrak et al., 2022). DDW consumption has a significant neuroprotective effect on rats exposed to a hypoxic environment by increasing the activity of antioxidant defense enzymes in the blood, thereby reducing the intensity of free radical oxidation and the expression of biomolecule peroxide modifications (Kravtsov et al., 2018). In line with these findings, a recent study by Kravtsov et al. also proved that DDW can prevent the development of oxidative stress in rat neural tissue under hypoxic conditions and ameliorate the survival rate of cultured neurons under glucose deprivation (Kravtsov et al., 2021). For Ehrlich ascites tumor-bearing BALB/c mice, reducing the deuterium concentration from 150 ppm to 85 ppm in the drinking water effectively reduced tumor-mediated oxidative stress in the liver, compared with that in the control group, which consumed tap water (Bayrak et al., 2022). In addition, DDW not only protects PC12 cells from H_2_O_2_-mediated oxidative damage but also promotes the repair of PC12 cells after oxidative injury, by attenuating H_2_O_2_-induced apoptosis, reducing intracellular ROS, increasing the activity of antioxidant enzymes, and modulating the expression of some proteins associated with the PI3K-Akt/PKB signaling pathway in PC12 cells (Wu et al., 2020).

### Effects of DDW on alleviating obesity, preventing diabetes and hypertension, detoxification, anti-inflammatory, and anti-radiation

In recent decades, obesity, diabetes, hypertension, and metabolic syndrome have become global public health issues. Recent studies have shown that DDW can inhibit the adipogenic differentiation of human adipose-derived stem cells (ADSCs) *in vitro* and alleviate diet-induced obesity and related metabolic impairments in a rat model, providing new insights into obesity treatment (Halenova et al., 2019; Zlatska et al., 2020). DDW can also promote insulin secretion, improve glucose and lipid metabolism, and prevent or delay insulin resistance and type 2 diabetes (Wu et al., 2020). With an optimal concentration between 125 and 140 ppm, DDW intriguingly enhances the effect of insulin on GLUT4 translocation and increases glucose uptake, thereby reducing the serum glucose, fructose amine-, and glycosylated hemoglobin (HbA1c) levels in streptozotocin (STZ)-induced diabetic rats (Molnár et al., 2021). Similarly, prospective clinical trials have indicated that continuous consumption of DDW at a concentration of 104 ppm for 90 days (1.5 L per day) remarkably reduced the deuterium concentration in the body and benefits patients with prediabetes or diabetes mellitus (DM) by altering several parameters related to metabolic syndrome, including insulin levels, glucose levels and high-density lipoprotein (HDL) levels (Somlyai et al., 2020; Somlyai et al., 2021a). In addition, DDW significantly reduced the levels of total cholesterol and triglycerides in normotensive Wistar Kyoto rats while increasing plasma insulin levels, which was associated with increased nitric oxide synthase (NOS) activity in the left ventricle. Moreover, in hypertensive rats, DDW can lower the increase in NOS activity regulated by iNOS (Rehakova et al., 2016).

The protective properties of the DDW were also confirmed by toxicological research, which indicated that DDW can effectively remove toxins and metabolic waste products from organisms (Syroeshkin et al., 2018). Previously, DDW was shown to alleviate the toxic effects caused by cadmium, chromium, manganese, carbon tetrachloride and gentamicin (Doina et al., 2008; Olariu et al., 2010; Ávila et al., 2012; Sergeevich et al., 2018). The possible reasons for these effects include the following: 1. DDW may alter the rate of biocatalytic processes in the cells of detoxification organs by reducing the activation energy of active enzyme groups, thereby achieving detoxification effects; and 2. DDW can also activate the non-specific defense systems through isotopic D/H exchange reactions (Sergeevich et al., 2018). Besides, the combination of DDW and *Mentha longifolia* essential oil has been shown to effectively prevent cecal ligation and puncture (CLP) induced sepsis through antioxidative stress, highlighting the synergistic anti-inflammatory effects of DDW (Rasooli et al., 2019). In addition, after exposure to 8.5 Gy of radiation, mice fed DDW (30 ppm deuterium) had a higher survival rate than mice fed normal distilled water, suggesting the radioprotective effect of DDW (Bild et al., 1999).

## Challenges and future directions

Undoubtedly, there are still many gaps in our knowledge regarding the effects of deuterium on living organisms, and the credible mechanisms by which deuterium affects biological systems need to be further unraveled and refined in the future. In response to the adverse and beneficial effects exhibited by deuterium enrichment, future research should focus on exploring an appropriate range within which an increased deuterium concentration will not cause toxic side effects or adverse impacts on the organism while also fully maximizing its potential beneficial effects. Second, although D_2_O, either in the form of DEW or DDW, has certain antiaging potential, considering the complexity of aging, where oxidative stress might be just one of the many mechanistic conditions involved (Demidov, 2007; Hammel et al., 2013), we cannot prematurely assert that the benefits observed from D_2_O do not arise from other mechanisms and only further validations could be used to resolve these issues. Last but not least, issues regarding DDW to exert its therapeutic effects require substantial resources and time to resolve, involving but not limited to: 1. Dose determination: the appropriate dosage of DDW for treating diseases needs to be established, as the optimal dose may vary depending on the type and course of disease, and individual factors such as age and weight; 2. Safety concerns: while DDW is generally considered safe and nontoxic, relevant evidence on the long-term effect of DDW consumption is scarce and a thorough investigation is needed; 3. Cost problem: the complex production process of DDW results in the relatively high price, which may be prohibitive for some patients or healthcare systems, thus the affordability of DDW treatment is needs to be addressed.

## Conclusion

Deuterium, a heavy isotope of hydrogen, is indispensable for living organisms, with different species varying in the tolerance to it. Both excess and depletion of deuterium can significantly impact life systems at the molecular, organelle, cellular, organ, and even whole organism levels. Deuterium-depleted water, as an important deuterium compound, has also shown tremendous potential in the adjuvant treatment of diseases such as tumors, depression, diabetes, and metabolic syndrome, opening new avenues for the treatment of these disorders and diseases. Nevertheless, the translation of the findings from concept to practice is far from straightforward. Before DDW can be widely implemented in medical practice, it is imperative to address certain issues, such as the optimal dosage, the safety of long-term application, and the cost concerns associated with DDW. Furthermore, additional clinical randomized controlled trials, ideally conducted in multicenter and prospective cohorts, are also needed to further validate its effectiveness.
